# Supplementary material for: Are carbon emissions trading and green financial instruments synergistic? -Comprehensive quantitative research based on content analysis
Source: PLoS One. 2024 Mar 7;19(3):e0298601. doi: 10.1371/journal.pone.0298601 (PMC10919601; doi:10.1371/journal.pone.0298601)
Supplement: S1 Data — (ZIP) [file pone.0298601.s001.zip › Data/地级市Z_F工作报告.zip!/─■╧─/╓╨╬└╩╨/└┤╫╘╣1⁄2╓┌║┼í╛┬φ┐╦╩2╛▌═°í┐_2019.docx]

各位代表：

现在，我代表市人民政府，向大会报告工作，请予审议，并请政协委员和列席人员提出意见。

一、2018年工作回顾

2018年，面对严峻的经济下行压力和繁重的改革发展稳定任务，在自治区党委、政府和市委的坚强领导下，在市人大、政协的监督支持下，市人民政府深入学习贯彻习近平新时代中国特色社会主义思想和党的十九大精神，切实践行新发展理念，坚持稳中求进工作总基调，紧紧围绕“转型追赶、高质量发展”主线，坚决打好“三大攻坚战”，大力实施“三大战略”中卫方案，全面落实“五个扎实推进”重点任务，全力以赴稳增长、促改革、调结构、惠民生、防风险，经济社会发展取得新成绩。预计地区生产总值增长6%，固定资产投资下降35%，地方一般公共预算收入同口径增长8.2%，社会消费品零售总额增长5.5%，城镇和农村常住居民人均可支配收入分别增长8%和8.5%。

一年来的主要工作成效是：

——发展质量稳步提高。实施创新驱动战略，增强科技创新能力，新增国家级高新技术企业3家，申请专利增长190.5%，万人有效发明专利拥有量2.05件。科技经费投入增长40%，研究与试验发展经费投入强度0.94%。实施特色农业提质增效行动，建立富硒产业标准化体系，成立硒产业发展公司，中卫被授予“中国塞上硒谷”称号。中宁枸杞获全国首个“农产品气候品质类国家气候标志”，“香山硒砂瓜”获批中国特色农产品优势区，“沙坡头苹果”获国家农产品地理标志。预计第一产业增加值增长3.8%。实施工业对标提升转型发展十大行动计划，工业技改投资增长16%。新培育认定“专精特新”中小企业15家、国家“两化”融合管理体系贯标试点企业4家，锦宁铝镁等4家企业分别获自治区工业企业对标标杆奖和进步奖，天元锰业获第五届中国工业大奖表彰奖。预计第二产业增加值增长5.4%。加快创建全域旅游示范市，成功举办环球旅游小姐世界总决赛、大漠黄河国际旅游节等节会赛事，接待游客760万人次，实现旅游收入62亿元，分别增长11.4%和15.7%。加快推进云计算和军民融合产业发展，成功举办首届云天大会，中卫被评为“最适合投资数据中心的城市和地区”，中卫军民融合产业园被认定为自治区级创业孵化示范园。预计第三产业增加值增长7.5%。

——改革开放深入推进。深化“放管服”改革，积极推进权力清单标准化建设，实行行政审批告知承诺制、工业园区项目“区域评”“代办制”，取消证照事项383项，“不见面”、网上办理率分别达到80%和88.9%。深入推进供给侧结构性改革，全面落实“去降补”各项工作，工业企业电力直接交易近122亿度，节约成本约8600万元，为企业减税14.3亿元。深化商事制度改革，新设立市场主体1.1万户，增长11.3%。加快农村集体产权制度改革，新成立土地股份合作社17家，办理“两权”抵押贷款2474户3.15亿元。加快国资国企改革，推行国有企业负责人薪酬试点。深化综合医改，公立医院改革取得积极进展，得到自治区肯定。全面落实自治区《深入推进内陆开放型经济试验区建设实施意见》，运行中欧、中俄国际货运班列20列，进出口总额达22.8亿元；加快畅通交通物流运输通道，海同高速公路建成通车，吴忠至中卫城际铁路通车测试，中卫南站黄河大桥、中卫至兰州客运专线等项目顺利实施。

——生态环境明显改善。从严从实抓好环保问题整改，中央环保督察转办件办结率91.8%。大力实施蓝天行动，取缔燃煤锅炉249台，清理整治“小散乱污”企业25家，建成洁净煤配送中心9个，优良天数达标率90.5%。大力实施碧水行动，全面落实河（湖）长制，扎实推进清河专项行动，重点河湖沟道环境质量明显改善，黄河中卫过境段水质达到Ⅱ类。大力实施净土行动，建立绿色防控示范区3个、农用地膜回收点39个，农用残膜回收利用率达85%以上。实施南部水源涵养林生态修复、北部防沙治沙、中部黄河城市过境段水生态治理等工程，完成营造林23万亩，森林覆盖率提高到13.9%。

——民生福祉持续增进。强化闽宁合作，全面推进产业扶贫、金融扶贫、就业扶贫、教育扶贫，实施农村饮水安全等重大扶贫工程，完成“十三五”2892户12016人易地搬迁任务，42个贫困村脱贫销号、18个贫困村出列、减贫2.8万人，贫困发生率从7.2%下降到3.95%。大力推动创业就业，新培育自治区级创业孵化示范区3家，新增就业9080人，农村劳动力转移就业15.4万人，城镇登记失业率3.6%。社会保障提标扩面，企业退休人员养老金人均提高135元，为8.3万名城乡居民人均月调增基础养老金20元，建档立卡户养老、医疗保险参保率分别达99.8%和100%。坚持优先发展教育，新改建幼儿园15所，改造义务教育薄弱学校17.8万平方米，建立覆盖各个办学层次的贫困学生资助体系，为1.5万名大学生发放助学贷款9796万元。大力发展医疗卫生事业，市中医医院等项目顺利推进，推行智能家庭医生签约服务，创建群众满意乡镇卫生院39个、社区（村）医疗机构109个，创建国家卫生城市通过自治区评审。着力繁荣城乡文化生活，举办广场文艺、惠民下乡演出1000余场次，成功举办第四届全国大漠健身运动大赛、2018国际沙滩排球中卫公开赛等赛事，圆满完成自治区成立60周年群众文艺演出任务。改善城乡人居环境，改造棚户区和老旧小区11万平方米，新建续建城市道路37条27.8公里，新建改建农村公路466公里，开工建设美丽小城镇3个、美丽村庄20个。开展扫黑除恶专项斗争，扎实推进“平安中卫”建设，公共法律服务体系全面建成。全力化解矛盾纠纷，严格落实领导值班接访和信访包案化解制度，中央巡视组信访转办件办结率96.1%。加强食品药品监管，严格落实安全生产责任制，事故起数和死亡人数“双下降”。开展民族宗教领域问题专项整治，深化民族团结进步创建，民族团结、宗教和顺的局面进一步巩固。

——政府效能不断提升。坚决贯彻自治区党委、政府和市委各项决策部署，坚持“三重一大”事项提交市委审定。认真执行市人大及其常委会的决定决议，建立向市人大常委会报告国有资产管理情况制度，自觉接受人大法律监督、政协民主监督和社会各界监督。办理人大代表建议17件、政协委员提案140件。全面推进依法行政，认真落实公众参与、专家论证、风险评估、合法性审查和集体讨论决定等制度，重要决策邀请“两代表一委员”、民主党派、群众代表参加，行政决策的科学化、民主化、法治化水平进一步提高。海原县国家级基层政务公开标准化规范化试点工作通过验收。强化政府债务管理，严格控制增量，积极消化存量，依法依规开展存量政府债务置换，债务风险总体可控。持之以恒纠治“四风”。积极推进审计全覆盖。“三公”经费下降19.1%。

同时，工会、共青团、妇女、科协、残疾人、红十字、慈善等事业健康发展，新闻出版、广播电视、统计调查、国防动员、双拥优抚、人民防空等事业稳步推进。

一年来，我们严格按照市委的决策部署，全力以赴抓落实，负重拼搏、攻坚克难，集中力量抓了一些事关全局的重点工作。我们隆重庆祝了自治区成立60周年，全面展示了中卫一甲子的辉煌历程，全市人民形成了“建设美丽新宁夏、共圆伟大中国梦”强大共识。中央代表团对中卫经济社会发展给予充分肯定。我们从无到有、从小到大，坚定不移发展云计算产业，依靠创新驱动推动转型追赶，通过延伸产业链逐步实现换道超车，中卫市变黄沙戈壁为创新发展新热土的典型经验被国务院通报表扬。我们立足市情，积极构建“一带两廊”空间发展布局，大力发展富硒产业、新型工业、军民融合、全域旅游、现代物流等新兴产业，推动产业结构转型、发展方式转变，正在闯出一条“腾笼换鸟”的发展道路。我们秉持以人民为中心的发展思想，始终坚持经济发展再困难、发展压力再大，民生投入不能降，在财政十分困难的情况下，仍然将70%以上的财力用于民生，解决了一大批群众最关心最直接最现实的民生问题。我们主动适应新常态，立规矩、转作风，狠抓政府效能提升，各级干部勇于担当、敬业奉献、务实创新，为全面建成小康社会积蓄了强大正能量。

各位代表，刚刚过去的一年，我市经济社会发展取得的成就来之不易，这是习近平新时代中国特色社会主义思想科学指引的结果，是自治区党委、政府和市委坚强领导的结果，是市人大、政协和社会各界鼎力支持的结果，是全市人民同心同德、真抓实干的结果。在此，我代表市人民政府，向为中卫经济社会发展付出辛勤劳动的全市人民，向给予政府工作大力支持的各位代表和委员，向各民主党派、工商联、无党派人士和人民团体，向驻卫部队和中央、区属驻卫单位，向所有关心支持中卫改革发展的社会各界朋友，表示崇高的敬意和衷心的感谢！

看到成绩的同时，我们必须清醒地认识到，全市经济社会发展和政府工作还存在一些问题和不足：经济下行压力仍然较大，项目接续储备不足，部分企业经营困难；转型追赶任重道远，新旧动能还处于“换挡期”；民生改善与群众期望仍有差距，基本公共服务均等化水平亟待提高；债务金融风险隐患不容忽视，脱贫攻坚仍需持续用力，污染治理和生态保护形势依然严峻；政府职能转变还不到位，一些工作人员作风不实，主动担当不够。需要说明的是，地区生产总值、固定资产投资等经济预期性发展目标没有实现，10件民生实事中有1件没有完成，这既有外部客观条件的制约，也反映出我们工作还存在着差距。对此，我们一定以对人民高度负责的态度，在今后的工作中全力以赴解决。

二、2019年主要目标和重点任务

今年是中华人民共和国成立70周年，是全面建成小康社会、实现第一个百年奋斗目标的关键之年，做好今年工作至关重要。从当前大局和趋势看，随着新时代改革开放再出发，随着新一轮西部大开发战略深入实施，随着“六稳”政策全面释放加力提效，支撑我市转型追赶、高质量发展的基本面没有变而且越来越好。我们一定要坚定信心、勠力同心，坚决贯彻中央、自治区和市委的决策部署，扎扎实实做好工作，努力向全市人民交上一份满意的答卷。

按照市委四届六次全会部署，今年政府工作的总体要求是：以习近平新时代中国特色社会主义思想为指导，深入贯彻党的十九大和十九届二中、三中全会及中央经济工作会议精神，统筹推进“五位一体”总体布局，协调推进“四个全面”战略布局，坚持稳中求进工作总基调，坚持新发展理念，坚持推动高质量发展，坚持以供给侧结构性改革为主线，坚持深化市场化改革、扩大高水平开放，持续落实自治区第十二次党代会和自治区党委十二届五次、六次全会以及市第四次党代会安排部署，继续打好“三大攻坚战”，接续实施好“三大战略”中卫方案，全面落实“五个扎实推进”重点任务，统筹推进稳增长、促改革、调结构、惠民生、防风险工作，保持经济持续健康发展和社会大局稳定，为与全国全区同步建成全面小康社会打下决定性基础，以优异成绩庆祝中华人民共和国成立70周年。

全市经济社会发展主要预期目标是：地区生产总值增长7%左右，规模以上工业增加值增长6%左右，固定资产投资实现恢复性增长，地方一般公共预算收入同口径增长8%左右，城镇常住居民人均可支配收入增长7.5%以上，农村常住居民人均可支配收入增长8%左右，社会消费品零售总额增长6%，居民消费价格涨幅控制在3%左右，城镇登记失业率控制在4.5%以内，研究与试验发展经费投入强度达1.1%，节能减排和环境质量改善完成国家和自治区下达任务。这一系列指标，既贯彻了中央和自治区的要求，又切合我市的发展实际；既考虑了速度和质量，又兼顾了需要和可能。这些目标，都不是轻轻松松就能完成的，需要我们自加压力、攻坚克难，尽最大努力争取最好结果。重点做好以下工作：

（一）围绕提升高质量发展水平，深入实施创新驱动战略，加快推动产业转型升级。依靠创新加快新旧动能转换，巩固“稳”的基础，积蓄“进”的力量，促进“质”的提升。

增强科技创新能力。立足产业基础，发挥特色优势，聚焦云计算、军民融合、精细化工、新能源、新材料、特色农业等领域，实施重点科技项目40个。加快沿黄科技创新改革试验区建设，推进创新要素集聚，建成国家高新技术企业10家、自治区科技型中小企业90家、自治区工程技术研究中心5个、各类创新创业平台55个，争创国家级农业高新技术产业示范区、自治区级高新技术产业开发区，与宁夏大学合作建设创新发展研究院。实施人才引育“三大行动计划”，完善人才评价、激励和服务保障体系，积极引进高层次人才、紧缺人才，培养用好本土人才、实用技术人才，持续强化中卫创新发展的人才保证和智力支撑。

做精做优特色农业。坚持创品牌、定标准、举龙头，大力发展“1+5”优势特色产业。加快建设富硒产业“一中心三基地”，建设富硒农产品基地12万亩、富硒高标准产业园16个。在品质品牌保护、市场营销上做文章、下功夫，着力打造中宁枸杞、中卫硒砂瓜、沙坡头苹果3个全国富硒农产品“单品冠军”。加快沙坡头区鼎腾、海原四季鲜等“五优”蔬菜基地建设，扩大示范富硒蔬菜种植，将“沙坡头”牌蔬菜打造成宁夏知名品牌。实施龙头带动工程，培育市级以上农业产业化龙头企业10家、示范合作社10个、家庭农场10家。推进农村一二三产业融合发展，推广“特色基地+龙头企业+电子商务”模式，打造产业化联合体5家。支持沙坡头区争创全国农村一二三产业融合发展先导区。扎实做好农田水利基本建设，加强农业技术推广服务，夯实现代农业发展基础。

做大做强新型工业。围绕新能源、新材料、冶金化工等重点产业，补链、延链、强链，推进产业向高端化、集群化方向发展。新材料产业坚持扩大增量和优化存量并重，支持引导今飞轮毂、锦宁巨科等企业开发新产品、增加新产能。重点建设中化锂电池正极材料、隆基硅单晶拉棒及切片生产线等技改项目。积极引进锰基复合材料项目，培育形成从金属锰、锰材料、复合材料到制造的产业链条，重点建设天元锰业2×350MW热电联产、粉煤灰深加工、40万吨石膏制酸等项目。新能源产业重点在提振企业发展信心、帮助企业开拓市场上下功夫，鼓励中卫银阳公司等企业走出国门开拓海外市场，力促国电投200MW等项目建成投产。加快推进中卫工业园区增量配电业务改革试点工作，有效降低企业用电成本，实现用电企业与新能源企业双赢。冶金化工产业坚持走高端、绿色、循环化发展路子，积极引导三元中泰等冶金企业加快建设余热发电项目，实现节能减排低碳循环发展，重点抓好瑞泰芳纶、华御合成高温导热油、紫光天化蛋氨酸关键工艺改造等项目建设，紧盯中化、苏化、重庆化医等企业开展产业链招商，加强上下游产业配套和各类产品互补，提高产业集聚发展水平。持续推进卫宁工业园区融合发展，宣和、镇罗、常乐等园区整合发展。提高工业园区规划建设水平，高质量完成工业园区规划修编，完善区域基础设施配套。深入实施“生态园区、绿色工厂”行动计划，着力打造生态优良、环境美好、运行有序、产业兴旺的工业园区。

做靓做活现代服务业。坚定不移、强力推进云计算、军民融合、全域旅游、现代物流发展。云计算产业在数据中心扩规、吸引更多企业落户上双向发力，力争把西部云基地打造成全国乃至世界云计算产业集聚区。加速亚马逊合作数据中心、中国移动数据中心扩容，加快美利云二期、中国联通、炫我科技、天云网络等在建数据中心进度，力促中国电信、万达信息、人民网等数据中心开工建设，争取国家部委、央企数据灾备基地和京东等企业落户中卫。加快推进国信安全网络创新应用基地建设。军民融合产业紧盯创建国家军民融合创新示范区目标，按照“一中心、四基地、一展会”的发展思路，加快推进达天飞艇、“宁夏一号”（钟子号）卫星和天线组阵等项目建设，力促卫星定标与真实性检验场、低空空域综合信息服务保障平台、空间碎片监测中卫基地等项目开工建设，吸引更多国内外军民融合企业在中卫集聚发展。全域旅游产业按照示范市创建三年行动计划，突出抓好沙坡头沙漠区、天湖等景区开发建设，加快推进旅游云轨、房车露营地等项目建设。进一步完善沙坡头、寺口子等景区基础设施，完成双龙石窟、天都山等3A级景区创建工作。加快乡村旅游发展，丰富旅游产品供给。深化“冬游三亚、漳州，夏游中卫”区域合作，继续开发旅游包机、旅游专列。办好大漠黄河国际旅游节、环球旅游小姐世界总决赛等赛事。力争全年游客数、旅游总收入增长12%以上。现代物流产业紧盯“物”的聚集和“流”的条件，着力将交通区位优势转化为产业发展优势。加快建设中卫迎水桥保税中心、中卫工业园区公铁物流园区，开工建设中国物流中卫物流园二期，引进8至10家3A、4A级物流企业，有效提升中卫物流园综合服务功能。加快海兴开发区物流产业发展，把海兴开发区打造成“龙头企业+物流+高端肉牛深加工全产业链”的农产品集散地。加强与青岛港、日照港等东部港口合作，开展铁路班列及多式联运合作，优化中卫运输结构。大力拓展货物包机业务，与顺丰丰鸟公司合作，开通中卫至固原货运航线，打造西北航空货运分拨中心。加强与传化集团、中国物流等物流龙头企业和物流园区战略合作，把中卫打造成全面开放、区域互通和低成本的综合物流集散地。

全力扩大有效投资。紧盯中央和自治区政策取向和资金投向，抓住机遇、精准谋划，加强脱贫攻坚、铁路、公路、机场、水利、能源、农业农村、生态环保、社会民生九大领域项目储备力度，形成符合中央、自治区发展战略、切合中卫实际的梯次项目库，增强可持续发展后劲。坚持投量、投向、投效并重，突出抓好70个重点项目，力争完成投资100亿元以上。加快推进一批项目，重点抓好保障性安居工程、恒大都市广场等在建项目进度，确保早建成、早投运。开工建设一批项目，加强协调服务，推动华润新能源呱呱山200MW风力发电等项目开工建设。坚持引资、引技、引智并重，加大精准招商力度，跟踪落实已签约项目，切实提高项目履约率、资金到位率和投产达效率。

各位代表，创新是引领发展的第一动力，没有什么要素比创新要素更有活力，没有什么人才比创新人才更具潜力，没有什么动力比创新驱动更为强劲。我们要始终依靠创新驱动发展，使中卫成为孕育财富的沃土、创造财富的沃土、创新创造者实现梦想的沃土！

（二）围绕筑牢高质量发展底线，深入实施脱贫富民战略，加快推进发展成果共享。牢固树立以人民为中心的发展思想，坚持尽力而为、量力而行，多办利民实事，多解民生难事，不断提高人民群众的获得感、幸福感、安全感。

坚决打赢精准脱贫攻坚战。围绕“两不愁三保障”，扎实推进打赢脱贫攻坚战三年行动，精准发力、精准施策，确保年内47个贫困村脱贫出列、2.72万贫困人口脱贫、海原县脱贫摘帽。狠抓特色产业促增收。紧盯22个乡镇73个深度贫困村，坚持“一乡一业”“一村一品”，精准发展优势特色产业，形成户户有增收项目、村村有致富产业。实施“四个一”示范带动工程，培育特色产业示范区10个、旅游扶贫示范村10个。巩固扩大华润“基础母牛银行”扶贫成效。加大金融扶贫，力争建档立卡贫困户扶贫小额贷款覆盖面达80%、贫困户均贷款5万元以上。发展壮大村级集体经济，力争收入5万元以上。完善基础设施补短板。加大项目资金投入，建成农村公路261公里。实施农村饮水安全巩固提升工程6处，受益32.7万人，贫困村自来水入户率达95%以上。进一步加大生态移民村环境整治，不断完善基础设施。改造危窑危房3500户，切实解决生态移民迁出区遗留问题，确保移民可持续发展。健全工作机制强保障。完善与漳州市的市级扶贫协作平台。加强扶贫资金监管，统筹整合有效使用，确保发挥最大效益。有效激发贫困群众内生动力，带动群众稳定增收，实现脱贫致富。严格贫困退出机制，对已脱贫的贫困村脱贫户摘帽不摘责任、不摘政策、不摘帮扶、不摘监督，有效防止返贫和产生新的贫困，切实巩固提升脱贫成果。

千方百计促进就业。落实完善各项就业政策，重点抓好高校毕业生、农民工、城镇困难人员、退役军人等群体就业，保证城镇新增就业0.7万人以上，农村劳动力转移就业13万人次。把创新、创业更好结合起来，争创国家级创业孵化示范园区1个、自治区级示范园2个。加强和改进就业服务，加大职业培训，启动建设市职业技能公共实训基地，在劳动密集型产业技能培训上采取有力措施，在农村妇女就业培训上采取有效举措，着力提高培训精准度，高质量完成精准脱贫能力培训9920人，争取就业一人、受益一家、影响一片。在构建和谐劳动关系上持续用力，健全工资支付监控、工资保证金等制度，加大工资清欠特别是农民工工资清欠力度，努力保障劳动者按时足额领取工资报酬。

推进基本公共服务均等化。深入学习贯彻全国和全区教育大会精神，落实立德树人根本任务，大力发展普惠性学前教育，推进义务教育优质均衡发展。加快职业教育产教融合步伐，与宁夏大学共同办好中卫校区。抓住实施“互联网+教育”的契机，着力扩大优质教育资源供给，推进素质教育迈上更高水平，让孩子们不仅“有学上”，还要“上好学”。加快“健康中卫”建设，加大医疗卫生投入，建成市中医医院等项目，继续改善市、县、乡医疗卫生机构设施条件和装备水平，健全村卫生室服务功能，建设“互联网+医疗健康”数据中心和示范园。不断深化医药卫生体制改革，推进县域医疗一体化改革试点，开展医保支付制度改革前期工作，推动优质医疗资源下沉，在解决百姓“看病难”“看病贵”问题上取得突破性进展。认真落实城乡困难群体“低保”等各项政策，持续做好社会保障提标扩面工作，建成投运市老年人服务中心，增加养老床位尤其是护理型养老床位供给，继续推进保障性住房建设，建成市残疾人康复中心、慈爱重度精神病患者康复中心等项目，让困难群体享受到更多社会关爱，生活得更有尊严。

推动文化繁荣兴盛。深入开展社会主义核心价值观宣传教育，坚定文化自信，增强文化自觉，弘扬主旋律，传递正能量，构筑共有精神家园。落实“1+9”工作机制，加快全国文明城市创建步伐。实施“十大文明行动”，深化文明村镇、文明单位、文明家庭、文明校园创建。实施文化惠民工程，建好用好村级综合文化服务中心、农村文化大院。广泛开展群众性文体活动，推动全民健身与全民健康深度融合。加强移风易俗工作，发挥好村民议事会、红白理事会、村规民约作用，常态化整治大操大办、高价彩礼、厚葬薄养等不良习俗。始终把意识形态工作摆在重要位置，牢牢把握意识形态主导权，树立中卫好形象，传播中卫好声音。

加强和改进社会治理。巩固农村、社区网格化管理成果，推动社会治理重心向基层下移。健全多元化矛盾纠纷调解和社会稳定风险评估机制，深化基层治理、源头治理，有效化解社会矛盾。深化“七五”普法，加快依法治市进程。深化扫黑除恶专项斗争，严厉打击各类违法犯罪。开展禁毒示范市创建活动，推进社会治安防控体系建设，维护社会和谐稳定。开展“安全生产基础年”建设活动，落实安全生产责任制，强化源头管控和安全准入，排查整治各类安全隐患，提高应急救援水平，坚决遏制重特大事故发生。加强食品药品监管。深化全民国防教育，做好退役军人管理服务，提升双拥共建质量。全面贯彻党的民族宗教政策，依法加强宗教事务管理，创建全国民族团结进步示范市。

各位代表，发展成果由人民创造，就应该由人民共享。我们必须紧盯困有所帮、学有所教、病有所医、劳有所得、老有所养、住有所居，持续用情、用心、用力，让中卫人民群众生活越来越幸福、越来越有盼头！

（三）围绕夯实高质量发展基础，深入实施生态立市战略，加快推进美丽中卫建设。把环保作为经济社会发展的高压线、生命线、保障线，坚决落实环保责任，努力使中卫的天更蓝、水更清、空气更清新。

打好污染防治攻坚战。加强大气污染防治。全面实施中卫市打赢蓝天保卫战三年行动，升级改造燃煤锅炉环保设施，城市建成区禁止新建35蒸吨以下燃煤锅炉，淘汰工业园区20蒸吨以下燃煤锅炉。改造钢铁、化工、铁合金等行业污染治理设施，加快“小散乱污”企业整治，推进工业污染物全面达标排放。优良天数达标率、PM10、PM2.5平均浓度达到自治区考核要求。推进水污染防治。打好黄河保卫战，深入开展城市黑臭水体治理，全面加强水源地保护。严格落实河（湖）长制，持续开展重点入黄排水沟治理，巩固整治成果。实施城镇污水处理提标改造三年行动，加快中宁第一污水处理厂提标改造等项目建设，确保重点入黄排水沟水质稳定达到Ⅳ类。深入推进土壤污染防治。启动净土保卫战三年行动，完成农用地土壤污染状况详查。坚决禁止秸秆焚烧。推广农用残膜回收利用技术，回收利用率达90％以上。实行城市垃圾分类处置，推进生活垃圾焚烧发电、餐厨垃圾无害化处理，努力为全市人民创造一个良好的生态环境。

加强生态保护和修复。持续实施自然保护区及重点区域恢复治理。理顺自然保护区管理体制机制，促进自然生态资源有效保护与合理利用。大力实施植绿增绿行动，加快建设引黄灌区平原绿洲绿网提升、南部水源涵养林建设、移民迁出区生态修复等工程，巩固天然林保护、禁牧封育、防沙治沙等成果，完成营造林17.6万亩。继续推行最严厉的封山禁牧政策。

推动绿色低碳发展。坚持生态优先，严格生态空间管控，严守生态保护红线。推动产业结构绿色转型，加快淘汰落后产能、化解过剩产能，完成自治区下达的目标任务。严格产业项目准入，从严落实“三线一单”管理。大力发展节能环保、清洁生产、清洁能源产业，推进污水处理、垃圾处置、土壤修复、多污染物协同控制等技术研发应用。实施好中卫市全国节水型社会创新试点项目，为全国推广总结经验。持之以恒抓好中央环保督察反馈问题整改落实，确保按期完成、圆满交卷。

各位代表，绿色是新时代最鲜明的特色、最亮丽的底色。我们要着力建设西部重要的生态安全屏障，筑牢中卫可持续发展基础，形成绿色生产方式和生活方式，让“风景”变成产业，让“美丽”成为生产力，走出一条生态优先绿色发展的新路子！

（四）围绕构建高质量发展格局，着力促进城乡区域协调发展，加快推动“一带两廊”建设。牢固树立全市“一盘棋”思想，认真落实“一带两廊”空间规划，有效统筹全域空间资源配置，实现经济社会健康可持续发展。

强化规划引领作用。全面落实《大力实施乡村振兴战略 加快推进城乡一体化发展意见》，完成“一带两廊”空间规划和4个中心城镇、11个特色小镇发展规划。建设沿黄生态经济带，在抓好工业、物流的基础上，突出抓好康养产业、功能农业、休闲旅游等经济效益好、带动能力强的产业，重点推进特色小镇建设，在产业融合发展上蹚出一条符合中卫实际的新路径。建设扬黄特色产业廊，突出抓好枸杞、硒砂瓜、苹果等兼备知名地域品牌、优质产品品牌的优势特色产业，打造一批优势主产区，叫响“富硒产品在中卫”，使我们的富硒产品走向全国、蜚声中外。建设脱贫富民产业廊，突出抓好草畜、马铃薯、小茴香等适宜产业、富民产业，依靠农业科技服务、强化产品深加工实现提质增效，将特色种养业的比较优势真正转化为经济优势、经济效益，助力农民增收、农业兴旺。

提升城市治理水平。以创建全国文明城市和国家卫生城市为抓手，持续开展城市“双修”，实施市区亮化、基础设施完善工程，改造老旧小区20个，新建、续建市政道路28条，提升城市形象。建设市区青少年运动中心广场，启动市博物馆展陈工程，拓展文化馆、图书馆、体育馆综合服务能力，提高城市品位。扎实推进绿色建筑工程，开展装配式建筑试点工作。完善智慧公交系统，加强公交车规范运营管理，着力解决交通拥堵、停车难等“城市病”。深化城市管理执法体制改革，促进城市管理法治化、智能化、精细化。

加快美丽乡村建设。落实乡村振兴战略，抓好中心城镇、特色小镇建设，加快公共服务向城镇延伸，促进特色产业向城镇集聚。保护城镇的特色风貌，彰显城镇的文化底蕴，使城镇成为拉动中卫发展的新引擎。以农村垃圾、污水治理和村容村貌提升为主攻方向，深入推进农村人居环境三年整治行动，建成美丽村庄20个、美丽小城镇3个，改造农村厕所4000户。围绕打通供水、供气的“最后一公里”，提升供电、信息网络的保障能力，加快优质公共服务资源向农村覆盖，让乡村更加宜居，让农民生活的更有品质。

各位代表，协调是持续健康发展的内在要求。我们必须按照“一带两廊”规划，坚持一张蓝图干到底，统筹抓好乡村振兴、城市建设、产业发展、公共服务、环境保护、基础设施，让中卫的城市、乡村成为文明进步、秀美富饶、安居乐业的美好家园，让城市与乡村各美其美、美美与共！

（五）围绕激发高质量发展活力，着力深化改革扩大开放，加快营造一流发展环境。在巩固、增强、提升、创新上下功夫，深化供给侧结构性改革，以更大的决心和力度，推进改革开放不断向纵深发展。

全面完成政府机构改革。严格按照《中卫市机构改革实施意见》，3月底前全面完成市县政府机构改革。通过改革，切实优化部门机构设置、厘清部门职责边界划分、理顺市区事权关系，为提高政府行政效率和行政效能提供保障。

大力优化营商环境。深化“放管服”改革，最大限度方便群众、更高效率服务企业。围绕审批服务便民、高效，深化“互联网+政务服务”“互联网+监管”，全面推行全程网上登记注册，实现“一门、一网、一次”受理办结。落实投资项目在线审批工作，规范和压缩项目审批时限，政府、社会投资项目审批时间压缩到40个工作日，企业开办时间压缩到3个工作日以内。全面落实自治区促进民营经济健康发展“20条”，更大力度减税降费，多措并举降低企业运营成本，激发民营企业投资动力，吸引更多企业来卫投资置业。

打好防范化解重大风险攻坚战。坚决落实中央“坚定、可控、有序、适度”的要求，尽职尽责做好风险防控工作。全面实行地方政府性债务限额管理和预算管理，积极稳妥化解存量债务，通过财政预算安排、置换地方政府债券等多渠道偿还债务。坚决打击违法违规金融活动和非法集资行为，切实维护金融市场稳定。强化“借债还钱”的风险意识，坚决杜绝超出财力承受范围、盲目铺摊子，既要为重点建设提供资金保障，也要为化解风险创造条件。

深化农业农村改革。加快农村土地制度改革，实行农村土地“三权分置”制度，落实集体所有权，稳定农户承包权，放活土地经营权，促进农村土地适度规模经营发展，新建土地股份合作社10个。完成农村集体经济组织成员身份界定、股权量化，有条件的村全部成立村集体经济合作社或村集体股份经济合作社。扩大农村产权确权登记颁证范围和交易范围，稳步推进农村产权抵押登记贷款试点工作，逐步扩大可抵押贷款产权覆盖面。加快农村水利改革，推进水权交易和农业水价综合改革，促进水资源节约高效利用。

构建更加开放发展格局。对接内陆开放型经济试验区建设，畅通交通物流通道，开工建设乌玛高速公路中卫段，加快推进中卫南站黄河大桥、中卫至兰州客运专线、国道338、常乐黄河公路大桥等工程，争取启动沙坡头机场和中卫火车站改扩建工程。吴忠至中卫高铁年内建成通车，圆全市人民的“高铁梦”。坚持高水平引进来，积极引进区内外企业来中卫发展；支持全方位走出去，推动中卫企业在参与国际国内竞争中实现自身发展。

各位代表，改革开放是当代中国发展进步的必由之路，好的发展环境才是最优惠的政策。我们要弘扬敢闯敢试、敢为人先的改革精神，逢山开路、遇水搭桥，把改革开放的旗帜举得更高更稳，以改革促开放，以开放促发展，让我们的发展环境像美丽的沙坡头一样，成为中卫最靓丽的名片！

三、建设人民满意的服务型政府

新时代新任务，对政府自身建设提出了新的更高要求。新的一年，我们承载着更大期盼，肩负着更大责任。必须把全面从严治党贯穿于政府建设始终，把“五个过硬”要求贯穿于队伍建设各方面，打造忠诚干净担当、为民务实清廉的公务员队伍，加快建设服务政府、责任政府、法治政府、廉洁政府。

加强政治建设。把政治建设放在政府自身建设的首位，把党的领导贯穿政府工作全过程，树牢“四个意识”，坚定“四个自信”，坚决做到“两个维护”。坚决贯彻自治区党委、政府和市委的决策部署，切实履职尽责。坚持学思践悟、知行合一，强化政府工作人员教育培训，学习新政策、掌握新知识、增强新本领，更好担负起推动中卫经济社会高质量发展的重任。

建设服务政府。始终把人民群众对美好生活的向往作为奋斗目标，紧扣经济社会发展实际，紧扣人民群众生产生活，大兴调查研究之风，多谋为民利民之策。坚持“实”字当头、“干”字为要，不摆花架子，不搞形式主义，沉下心来、扑下身子，增强创新意识、实干意识和服务意识，争做实干家，真心实意为企业和群众排忧解难。

建设责任政府。把提高政府效能作为政府自身建设的重中之重，扎实开展“党政机关作风建设年、干部队伍素质提升年”活动，坚决整治庸懒散软拖等不作为、慢作为、乱作为现象，推动政风行风持续好转。健全政府运行机制，确保中央和区、市决策部署落地见效。加大政务失信行为惩戒力度，不断提高政府执行力和公信力。落实好干部容错纠错激励机制，旗帜鲜明鼓励干部干事创业。

建设法治政府。全面贯彻《法治政府建设实施纲要》，巩固行政执法“三项制度”改革成果，严格按法定权限和程序履行职责。全面推进政务公开，落实政府新闻发布制度，完善政府法律顾问制度，推动决策科学化、民主化、法治化。自觉接受市人大及其常委会的法律监督和工作监督、市政协的民主监督，认真听取民主党派、工商联和社会各界意见建议，不断提高人大代表建议和政协委员提案办理质量。

建设廉洁政府。市政府一班人以身作则、以上率下。全面落实党风廉政建设主体责任和领导干部“一岗双责”，健全廉政风险防控制度，加强对重点领域、关键环节、重要岗位权力运行的监督制约。加强审计监督，坚决惩治各类腐败行为。严格执行、较真落实中央八项规定及其实施细则，坚决纠正“四风”，群众反映强烈的问题都要认真对待，损害群众利益的行为都要坚决纠正，以政府的“清廉指数”提升人民的“幸福指数”！

各位代表，伟大的新时代，呼唤着伟大的实践。中卫人民正满怀豪情地踏上新的征程。“我们都在努力奔跑，我们都是追梦人”。让我们更加紧密地团结在以习近平同志为核心的党中央周围，在自治区党委、政府和市委的坚强领导下，不忘初心、牢记使命，振奋精神、实干兴卫，为实现经济繁荣民族团结环境优美人民富裕、确保与全国全区同步建成全面小康社会的目标而不懈努力，谱写“建设美丽新宁夏、共圆伟大中国梦”的中卫篇章，以优异成绩向中华人民共和国成立70周年献礼！
